# Characterization of pea seed nutritional value within a diverse population of *Pisum sativum*

**DOI:** 10.1371/journal.pone.0259565

**Published:** 2021-11-04

**Authors:** Gokhan Hacisalihoglu, Nicole S. Beisel, A. Mark Settles

**Affiliations:** 1 Department of Biological Sciences, Florida A&M University, Tallahassee, Florida, United States of America; 2 Horticultural Sciences Department, University of Florida, Gainesville, Florida, United States of America; University of Melbourne, AUSTRALIA

## Abstract

Micronutrient malnutrition is a global concern that affects more than two billion people worldwide. Pea (*Pisum sativum*) is a nutritious pulse crop with potential to assist in tackling hidden hunger. Here we report seed ionomic data of 96 diverse pea accessions collected via inductively coupled plasma mass spectrometry (ICP-MS). We found a 100 g serving of peas provides the following average percent daily value for U.S. recommendations: 8% Ca, 39% Mg, 73% Cu, 37% Fe, 63% Mn, 45% Zn, 28% K, and 43% P. Correlations were observed between the majority of minerals tested suggesting strong interrelationships between mineral concentration levels. Hierarchical clustering identified fifteen accessions with high-ranking mineral concentrations. Thirty accessions could be compared to earlier inductively coupled optical emission spectrometry (ICP-OES) data, which revealed significant differences particularly for elements at extreme low or high levels of accumulation. These results improve our understanding of the range of variation in mineral content found in peas and provide additional mineral data resources for germplasm selection.

## Introduction

Micronutrient nutritional quality is important in addressing the global issue of “hidden hunger”. Hidden hunger, or micronutrient malnutrition, affects over two billion people worldwide [1], and can lead to a variety of health complications. Diets deficient in micronutrients raise the risk for blindness, lower IQ, and immune deficiencies [2]. Strategies for reducing the prevalence of micronutrient malnutrition have primarily focused on encouraging people to take dietary supplements, to increase diversity in their diets, and to fortify foods during processing [2]. While these strategies positively impact the issue of hidden hunger, success has been limited [2]. Biofortification improves the nutritional content in agricultural crops through breeding or biotechnology and is a promising strategy to help tackle micronutrient malnutrition [3].

Breeding efforts to biofortify crops require data on variation in nutritional content within the natural diversity of target crop species. Ionomics is the study of the elemental composition of biological organisms [4] and is an effective approach to analyze the comprehensive mineral content of existing plant germplasm. Profiling ionomes of germplasm collections has given insight on the potential range of elemental composition for multiple crop species such as maize [5] and pulses [6–11]. There are several spectrometry methods used to study ionomes in crops, such as x-ray fluorescence, inductively coupled plasma mass spectrometry (ICP-MS), inductively coupled optical emission spectrometry (ICP-OES), and atomic absorption spectrophotometry [12–14]. In recent years, ICP-MS has gained popularity, because the technique analyzes smaller sample sizes in less time and has higher sensitivity for elemental detection compared to other methods [12, 13].

Pea (*Pisum sativum*) is the third most important food legume in the Fabaceae family [15]. Peas are grown worldwide, incorporated into human diets as fresh, processed, or dried vegetables, and also used as a forage crop for animals. Pea production levels are similar to plantains and beans [15]. Domestication occurred over 7,000 years ago in current Turkey, making peas one of the oldest cultivated plant species [16]. Pea seeds are excellent sources of protein, dietary fiber, and mineral nutrients [17, 18]. Consumption of pulses, including peas, can help reduce risk of cancer and cardiovascular disease [19, 20]. Additionally, including peas in the diet can help manage diabetes by regulating blood glucose and insulin levels [19]. Pea seed protein is unlikely to cause allergenic reactions and is easily digestible [21]. The health benefits associated with eating peas, and the fact that they are already popularly consumed, makes them a prime candidate for biofortification efforts to reduce the prevalence of hidden hunger.

Shifting human diets from animal to plant protein sources can also help reduce greenhouse gas emissions from food production. The nutrition available in peas can serve as an alternative to meat or dairy, particularly when phytate levels are reduced in the seed [22, 23]. Peas, like other pulse crops, have a symbiotic relationship with rhizobia bacteria allowing the crop to fix nitrogen and be grown without the application of nitrogen fertilizer [19, 24]. Pea is also considered more sustainable among pulse crops, because it has a high water use efficiency compared to other pulse species [25]. However, rising CO_2_ levels negatively impact pea seed quality by reducing Fe, Zn, and protein concentrations [26]. Therefore, it is imperative for plant breeders to include nutritional quality selections within pea germplasm to increase mineral nutrients to mitigate the impacts of climate change.

Multiple studies have characterized micronutrient composition in pea germplasm in order to identify germplasm that can be used in biofortification. Coyne et al. [27] surveyed over 400 USDA accessions of *P*. *sativum* with ICP-OES using seeds from greenhouse grown plants. These data have been used to find markers associated with levels of Ca, Cu, Fe, Mg, Mo, Ni, and P [11, 12]. ICP-OES has also been used to survey 152 *P*. *sativum* accessions from Turkey [28]. ICP-MS was used as a reference method to develop calibrations for X-ray fluorescence spectroscopy from 153 pea accessions in the University of Saskatchewan pea breeding program [29]. Smaller collections of *P*. *sativum* have been analyzed for mineral content with a variety of methods [14, 30–34]. These studies compared 4–10 varieties in multiple field environments. Although different field environments have impacts on seed mineral composition, the replicated field trials also showed significant genetic effects on Ca, Cu, Fe, K, Mg, Mn, P, and Zn levels in pea seeds.

Here we report seed mineral composition of 96 pea accessions from the USDA germplasm collection as determined by ICP-MS from field grown plants. The seed ionomes were examined for relationships to seed protein, oil, and weight using analytical data from the same seed lots reported in a previous study [35]. The accessions profiled include 10 accessions that were analyzed by ICP-OES as well as 20 single plant selections derived from accessions profiled with ICP-OES [27]. These data allow a comparison of methods for quantifying micronutrients. The interrelationships among seed nutrient concentrations and percent daily value (% DV) of the nutrients were examined to identify accessions that could be used to boost mineral element accumulation in pea seeds.

## Materials and methods

### Pea material

A collection of 96 diverse global accessions of pea were selected from USDA National Germplasm Center (Pullman, WA) (S1 Table). Descriptive statistics for each nutrient and accession were determined using the average of the ICP-MS results from three biological replicates (288 seeds in total). These accessions were selected based on broad geographic sources, phenotypic diversity, and availability at the time of seed order. Each accession was grown at one of two field sites in Pullman, WA. The Central Ferry farm has Chard silt loam soil, elevation of 198 m, and sub-surface drip irrigation is used during the active growing season. The Pullman Whitlow farm has heavy soils including Palouse and Palouse-Thatuna silt loam, elevation of 790 m, and fields are dryland managed. At both locations, Treflan (Dow Chemical) was used as an herbicide, Warrior (Syngenta) was used as an insecticide, and fields were fertilized with ammonium thiosulfate post-planting and pre-emergence.

### Determination of elemental analysis

Seed elemental concentrations were quantified by Waters Agricultural Labs Inc. (Camilla, GA, USA). Minerals were analyzed by open vessel wet digestion using an inductively coupled argon plasma spectrometer (ICAP, DigiBlock 3000 ICP-MS). Seeds were dried at 80°C in an oven overnight and then ground in a Wiley mill. A 0.5 g sample was mixed with 5 mL concentrated nitric acid and incubated at 95°C for 90 min. Then, 4 mL of 30% H_2_O_2_ was added to each tube and incubated at 95°C for 20 min. Each sample was cooled for 2 min, brought to 50 mL with distilled H_2_O, and mixed well. Samples were transferred to ICP tubes for analysis in accordance with the manufacturer’s specifications. The ICP-MS was calibrated using distilled H_2_O as a blank, standards of pure elements mixed to known concentrations, and two maize reference samples.

### Estimation of nutritional value

The nutritional value of pea seeds was estimated for a 100 g dry weight serving portion. Information regarding the current DV recommendations was obtained from the Food and Drug Administration (FDA) (https://www.fda.gov/food/new-nutrition-facts-label/daily-value-new-nutrition-and-supplement-facts-labels). Protein content for the 96 seed lots was from a recent publication [35]. Percent DV was calculated using the following formula:

$$\%\;DV=100\times \frac{mg\;nutrient\;in\;serving}{mg\;recommended\;DV}$$

### Statistical analysis

Descriptive statistics for each nutrient and accession were determined using the average of the ICP-MS results from three biological replicates. The triplicate averages were analyzed both as standardized scores and raw values. Violin plots were generated using the geom_violin function within the R-package ggplot2 [36] using standard scores. Correlation scatterplots of selected seed trait relationships were constructed using the raw triplicate averages. Hierarchical clustering of the DV estimates was completed with Morpheus (https://software.broadinstitute.org/morpheus/) using options for Euclidian distance and complete linkage.

## Results

### Variation in pea seed mineral concentration

**Fig 1:**
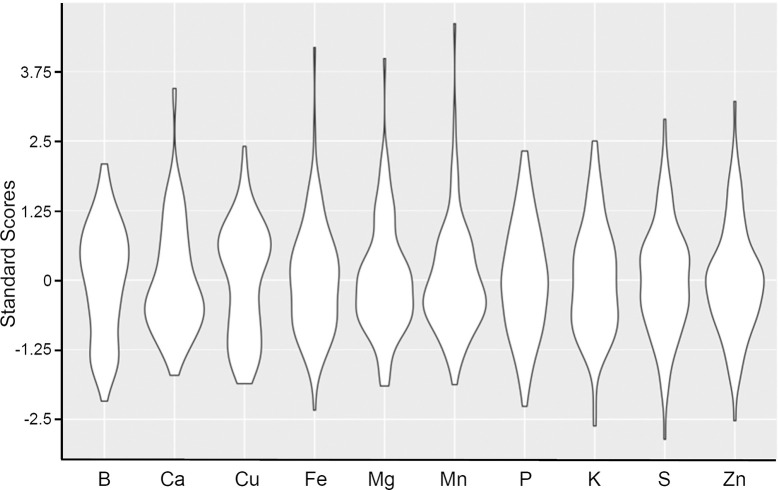
Violin plots displaying the distribution of standard scores for concentration of each element.

For the 96 pea accessions analyzed, K, P, S, Mg, and Ca were detected in the mg/g range of concentrations, while Fe, Zn, Mn, B, and Cu were detected in the μg/g range of concentrations (Table 1). K and P were the most abundant with average concentrations above 5 mg/g. B and Cu were the least abundant elements with average concentrations below 10 μg/g. Based on the coefficient of variation (CV, defined as the ratio of the standard deviation to the mean) for each element, Cu and Ca showed the largest variation in mineral content within these pea accessions. There was a 5.4-fold range of Cu concentration, and 4.7-fold range of Ca concentration (Table 1). Cu is at the lowest concentration of all elements measured with an average concentration of 6.6 μg/g, and it is possible that the variation observed is due to measurement error. However, the standard deviation of repeatability (SDR) for Cu is only 5% of the average concentration. By contrast, Ca accumulates at a much higher level of 1.05 mg/g average concentration, which is approximately 1% of total seed weight. These descriptive statistics suggest the variability is driven through biological sample differences instead of measurement error. The most stable mineral concentrations were K, S, and Mg with the CV ranging from 0.09–0.1 (Table 1). The SDR was less than 4% of the average value for all minerals, except Cu, indicating that the mineral content reported in S1 Table are accurate estimates of composition for each accession. Fig 1 shows violin plots of standard scores for each element to visualize the distributions within the germplasm sampled. B, Ca, and Cu have more bimodal distributions, while Fe, Mg, and Mn have a subset of accessions with extreme high accumulation.

**Table 1:** Descriptive statistics of seed mineral concentrations of 96 diverse pea accessions.

Units	Element	Avg	SD	Min	Max	SDR	CV
mg/g	K	13.36	1.24	10.11	16.5	0.13	0.09
	P	5.32	0.86	3.38	7.31	0.05	0.16
	S	2.48	0.23	1.81	3.16	0.02	0.09
	Mg	1.62	0.16	1.32	2.24	0.01	0.10
	Ca	1.05	0.34	0.49	2.22	0.02	0.32
μg/g	Fe	67.49	8.80	46.94	104.3	1.35	0.13
	Zn	49.70	9.11	26.70	78.94	1.00	0.18
	Mn	14.43	2.48	9.78	25.84	0.31	0.17
	B	9.69	2.01	5.31	13.89	0.38	0.21
	Cu	6.60	2.34	2.25	12.23	0.35	0.35

Avg, average; SD, Standard deviation; Min, minimum; Max, maximum; SDR, standard deviation of repeatability; CV, coefficient of variation.

### Relationship between different nutrients in seeds

**Fig 2:**
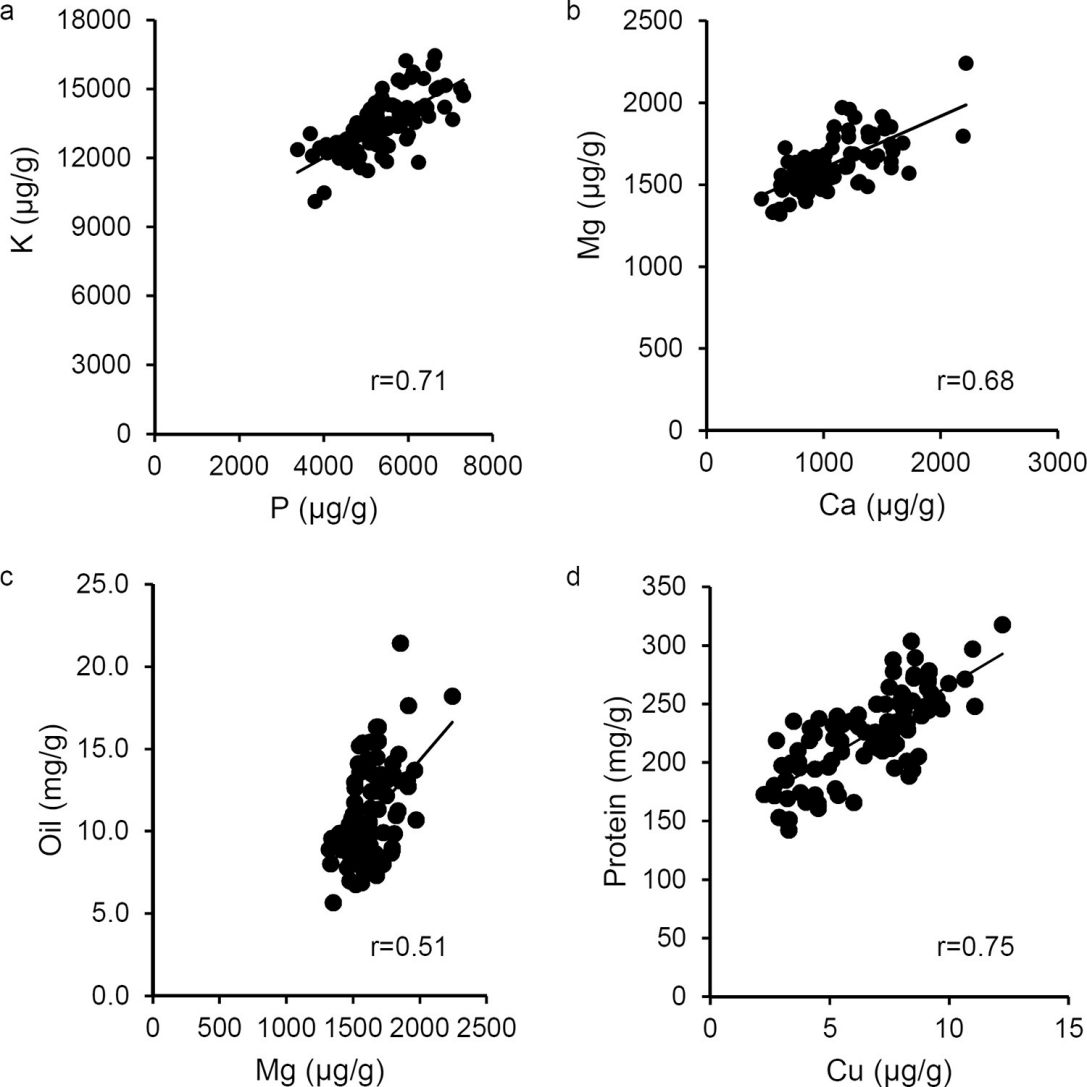
Examples of correlated seed traits. Each scatterplot shows a linear regression trend line and reports the correlation coefficient. All correlations shown are significant at p<0.01. (a) P and K. (b) Ca and Mg. (c) Mg and oil. (d) Cu and protein.

Positive correlations were observed between 24 pairs of element levels (Table 2). Fe had the fewest correlations with statistically significant positive relationships observed for Zn, Mn, and S. Both Mg and Mn showed the highest number of significant relationships with seven of nine other elements. However, most of the correlation coefficients were relatively low with only three pairs of elements (B-Cu, Ca-Mg, and K-P) showing correlation coefficients above 0.5. K and P had the highest correlation coefficient (r = 0.71, Fig 2A) with Mg and Ca having a slightly lower correlation coefficient (r = 0.68, Fig 2B).

**Table 2:** Seed trait correlation matrix.

Trait	Ca	Cu	Fe	Mg	Mn	P	K	Zn	S	Weight	Protein	Oil
**B**	**0.35**	**0.60**	0.06	**0.26**	**0.43**	0.07	0.04	-0.12	0.10	-0.03	**0.44**	0.16
**Ca**	-	**0.44**	-0.10	**0.68**	**0.37**	**0.22**	-0.05	-0.13	-0.07	-0.15	**0.42**	**0.45**
**Cu**		-	0.10	**0.39**	**0.24**	0.01	-0.11	-0.19	0.16	-0.09	**0.75**	-0.01
**Fe**			-	0.14	**0.37**	-0.09	0.07	**0.43**	**0.30**	-0.07	0.13	0.05
**Mg**				-	**0.47**	**0.42**	**0.38**	0.12	**0.32**	-0.09	**0.37**	**0.51**
**Mn**					-	0.13	0.16	**0.34**	**0.25**	0.01	**0.25**	**0.30**
**P**						-	**0.71**	**0.28**	**0.26**	0.16	0.10	**0.38**
**K**							-	0.18	**0.40**	**0.21**	-0.05	**0.45**
**Zn**								-	**0.29**	-0.15	0.02	0.09
**S**									-	0.06	**0.32**	0.04
**Weight**										-	**-0.24**	-0.17
**Protein**											-	0.12

Correlations with p<0.05 are bolded.

Additional trait data were obtained for the 96 seed lots analyzed in this study [35]. Published seed traits included seed weight, protein concentration, and oil concentration. These traits showed significant correlations with eight of the ten mineral levels (Table 2). Seed weight was positively correlated with K content, but no other minerals tested. Oil content was correlated with five individual mineral nutrients in pea seeds including: Ca, Mg, Mn, P, and K. Protein content was correlated with seven minerals including: B, Ca, Cu, Mg, Mn, and S. The largest correlation coefficients were observed between oil and Mg (r = 0.51, Fig 2C) as well as protein and Cu (r = 0.75, Fig 2D).

### Nutritional value of pea seeds

**Fig 3:**
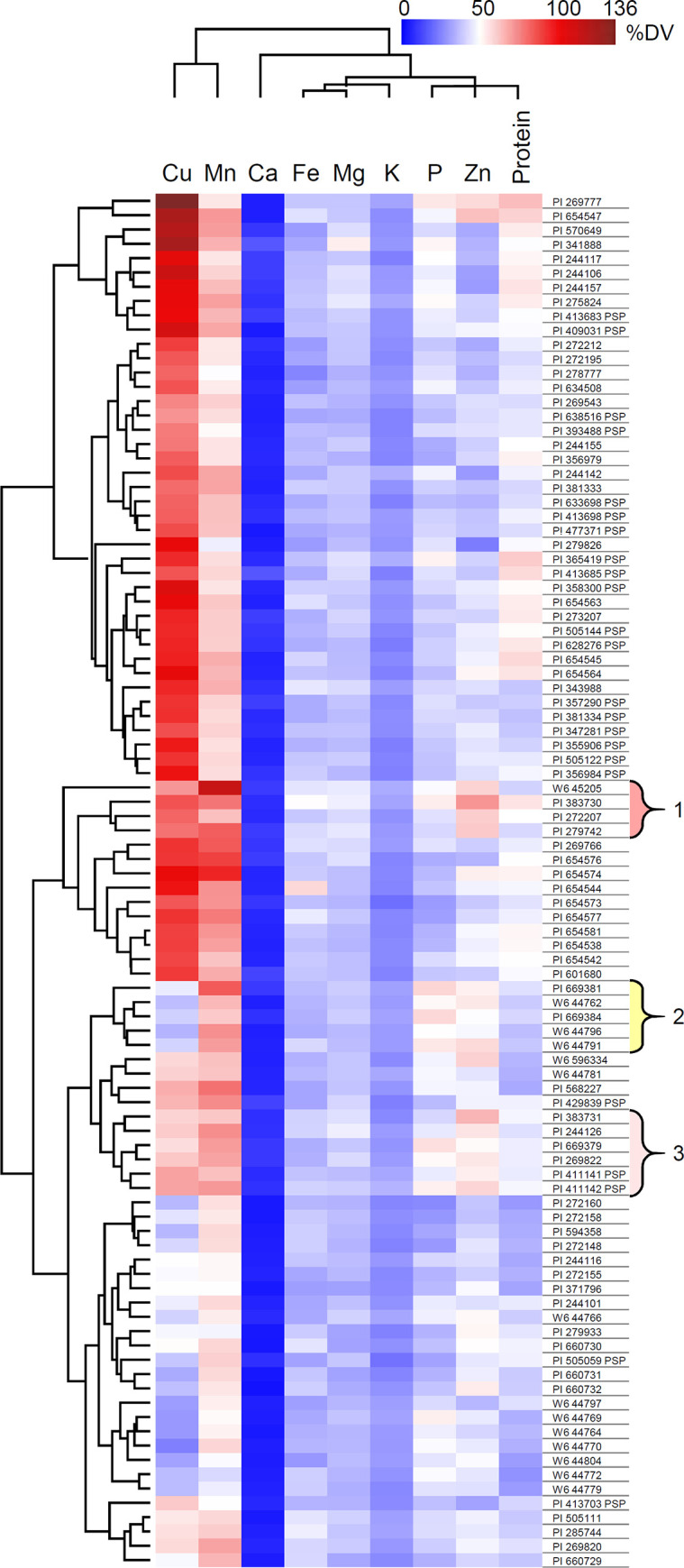
Heat map showing hierarchical clustering of the percent DV for a 100 g serving of each pea accession. Clusters of accessions with higher levels of mineral nutrients are numbered and indicated by braces. USDA-GRIN accession numbers are given for each accession.

Biofortification efforts should consider mineral variation in terms of human nutritional values in order to identify nutrients that could be breeding targets. Table 3 shows the FDA DV percentage averages and range for a 100 g serving of dried pea seeds based on a 2,000 calorie diet. Both Cu and Mn are abundant in the majority of accessions analyzed. Ca levels are universally low relative to the DV. The FDA does not provide DV for B and S, and data for these elements was not included in this analysis. Hierarchical clustering of the DV levels identified three clusters of accessions that have higher levels of Mn, Zn, and P (Fig 3). Cluster 1 includes four accessions: 250, Centrali-Sibiricum, Tiny Tim, and Ctirad, that have higher levels of Cu along with increased Mn, Zn, and P. Cluster 2 includes five accessions: Ai Zi Wan Dou, Ban Wan Dou, Lu Cai Wan, cri-1 af, and cri-1 st, that have lower levels of Cu with other minerals increased. Cluster 3 contains six accessions: 251, DELI, PERFECTION, cri-1, PANIA, and PATEA, that show an intermediate Cu accumulation phenotype.

**Table 3:** Mineral nutritional content of 100 g pea seeds as a DV percentage for a 2,000 calories human diet.

Mineral	Ave.	Min.	Max.
Cu	73	25	136
Mn	63	43	112
Zn	45	24	72
P	43	27	58
Mg	39	31	53
Fe	37	26	58
K	28	22	35
Ca	8	3	17

The FDA does not give DV recommendations for B or S.

### Comparison of ICP-MS and ICP-OES datasets

**Fig 4:**
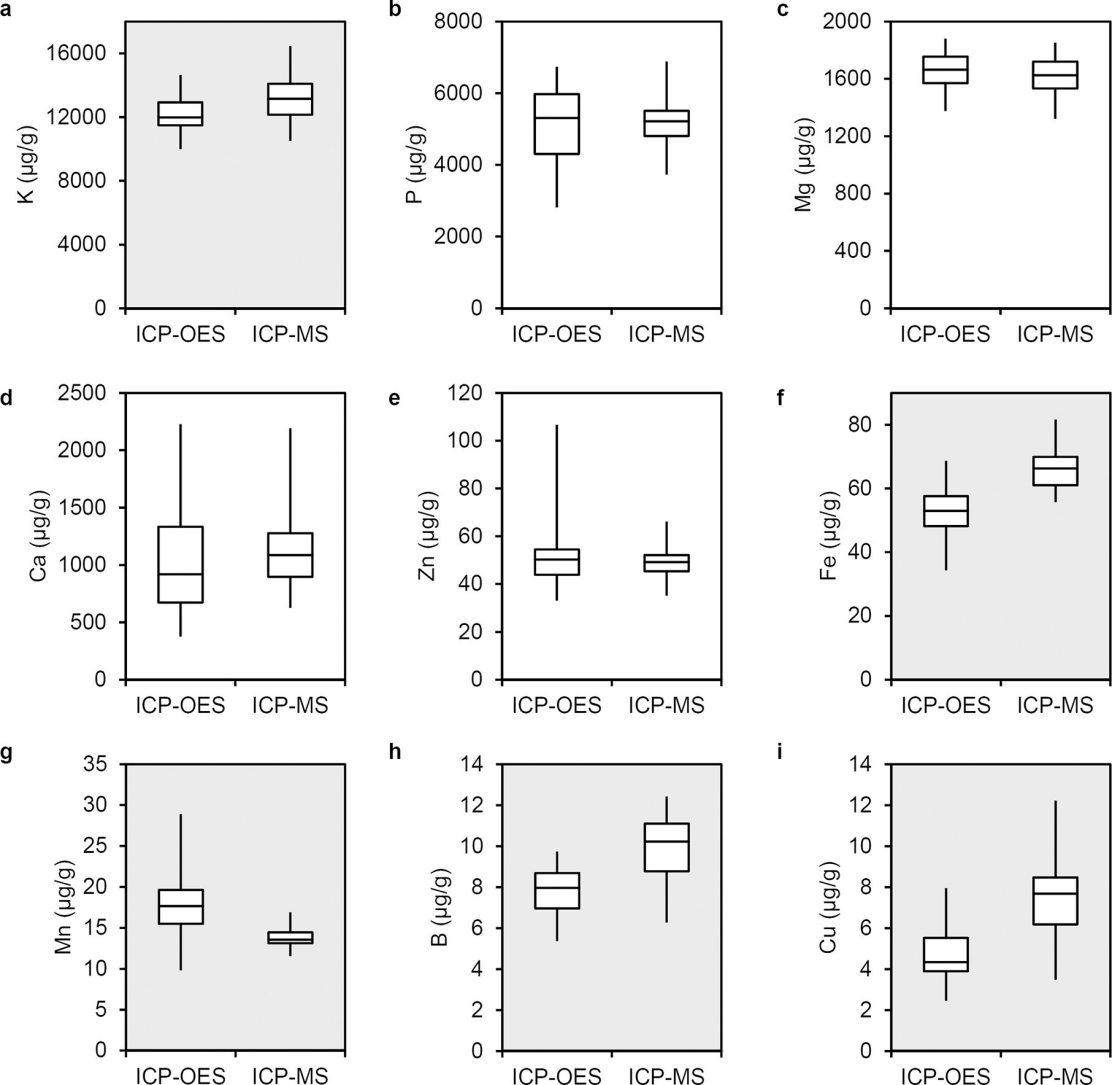
Boxplots comparing concentration data of elements from 30 pea accessions analyzed in this study with ICP-MS to prior ICP-OES data [26]. Plots with a gray background show significant mean differences with p< 0.01 for a paired Student’s t-test. Plots are ordered by descending average concentration: (a) K, (b) P, (c) Mg, (d) Ca, (e) Fe, (f) Zn, (g) Mn, (h) B, and (i) Cu.

Data previously collected by Coyne et al. [27] using ICP-OES was compared to ICP-MS data for 30 overlapping accessions in this study. Ten of the accessions are identical and 20 accessions derived from single plant selections from accessions that were profiled by ICP-OES. Table 4 reports correlation coefficients between the 30 accessions with genetic relationships. Ca and Mg were the only statistically significantly correlated minerals (r = 0.48 and 0.53, respectively). Although non-significant, negative relationships were observed for Cu, Fe, and Zn. Environmental variation is likely to have an impact on seed mineral composition, and it is also possible that sensitivity differences in the ionomics technologies could lead to differences in mineral traits. Most of the minerals that accumulate to low concentrations including Fe, B, and Cu had higher mean values measured by ICP-MS compared to ICP-OES (Fig 4). ICP-OES data also showed more variance for P, Ca, Zn, and Mn. Paired Student’s t-tests were used to determine minerals that showed mean differences in mineral composition between these technologies and environments. Significant differences (p<0.01) were observed for five of nine elements: K, Fe, Mn, B, and Cu (Fig 4A, 4F–4I). All of these significantly different elements accumulate at either the highest or lowest ranges of mineral concentration.

**Table 4:** ICP-MS and ICP-OES correlation coefficients with p<0.05 bolded.

Trait	Correlation coefficient
**B**	0.15
**Ca**	**0.48**
**Cu**	-0.14
**Fe**	-0.2
**Mg**	**0.53**
**Mn**	0.26
**P**	0.2
**K**	0.35
**Zn**	-0.27

## Discussion

The mineral concentration ranges observed in our survey are consistent with ranges reported for other *P*. *sativum* accessions suggesting that the nutrient levels in this study can be compared to other surveys of mineral composition [27–29]. Mineral nutrient values have not been reported for approximately ⅔ of the *P*. *sativum* accessions analyzed in this study. Similar to these prior surveys the most abundant elements observed were K and P, and the least abundant elements were Cu, B, and Mn.

The U.S. recommended DV for adults and children four years of age and older based on a 2,000 calorie diet are: 1,300 mg for Ca, 420 mg for Mg, 0.9 mg for Cu, 18 mg for Fe, 2.3 mg for Mn, 11 mg for Zn, 4,700 mg for K, and 1,250 mg for P. Relative to DV, Ca content is low in peas, therefore Ca is an important target for future biofortification efforts. When peas are cooked before consumption, both Ca and Mg levels are reduced [20]. All the statistically significant correlations between mineral concentrations were positive. The 0.68 correlation coefficient between Ca and Mg is especially interesting because these elements are also positively correlated with six other elements that accounted for more than 40% of the significant correlations observed: B, Cu, Mn, P, K, and S. The interconnections suggest that nutritional content in peas could be improved in general by targeting either Ca or Mg. Although the effect of Ca biofortification in peas is not reported, higher levels of exogenous Ca provide seedlings increased tolerance to salinity stress [37]. Moreover, there is evidence that Ca can show tissue specific accumulation suggesting any negative impacts of Ca biofortification on plant vigor could be genetically dissected if needed [38].

By contrast, Cu is already close to or above recommended DV. Cu is needed in the diet in trace amounts (DV = 0.9 mg/day), but excess Cu can be toxic and the tolerable upper intake level for Cu for adults is estimated to be 7–10 mg/day [39–42]. Roughly half (n = 49) of the accessions included in this study contain over 80% of the DV for Cu in a single 100 g serving, with fourteen of those accessions containing more than 100% of the DV. The maximum amount of Cu detected in a single 100 g serving of peas was only 1.2 mg. While this is well under the tolerable upper intake level, there is no need to further increase Cu in peas.

Cluster analysis of the mineral nutrients revealed accessions that had higher concentrations of Mn, P, and Zn minerals. Cluster 1 had the highest levels of Cu at ~80% DV for a 100 g serving. Cluster 2 generally had lower levels of the enriched minerals. Cluster 3 accumulated Cu to ~60% DV. These data suggest that some pea accessions may have genetic variation that can uncouple Cu levels from other minerals that require biofortification.

It is also possible that environmental differences are responsible for the clusters of higher mineral accumulation. This study contains seed sourced from single environments using two distinct soil types and irrigation methods. Prior replicated field trials with 4–10 genetic varieties have shown environmental effects for most of the minerals analyzed [14, 32–34]. However, multiple observations suggest that genetics contributes to the clusters with higher mineral content. First, Cu, Mn, P, and Zn are generally poorly correlated in field trials [34]. Second, when environmental effects have been estimated by variety, environmental effects on Cu, Mn, P, and Zn do not covary. In one field study, Cu and Zn were negatively correlated to Mn or P between field environments [14]. Consequently, increased levels of all four of the elements would not be expected if field environment was the primary driver of the seed composition differences observed. Third, when all three higher accumulating clusters are combined, there are clusters of varieties that appear to originate from individual breeding programs, including: cri-1, cri1 st, and cri-1 af (California, United States); PANIA and PATEA (Washington, United States); AI ZI WAN DOU and LU CAI WAN (Guangxi Zhuangzu Zizhiqu, China); 250 and 251 (Turkey). Although not conclusive, these observations suggest that genetics is likely to drive part of the higher accumulation in the clusters identified.

It should be noted that the present study did not investigate the bioavailability of the minerals tested in peas. Previous studies have found that certain minerals, primarily Fe and Zn, are not readily bioavailable in mature pea seeds due to high levels of the anti-nutrient phytate [43]. Recent work with low phytate pea lines showed that lower phytate concentration increases the bioavailability of Fe [23]. Our data identify additional germplasm that could provide high Fe, Zn, and Ca for biofortification breeding with existing low phytate germplasm.

Prior ICP-OES mineral concentration data were available for 30 USDA core pea germplasm accessions analyzed in this study [27]. The entire ICP-OES USDA core pea germplasm mineral concentration dataset has been analyzed in several genome wide association studies to identify markers that could be used for biofortification [10, 11]. The ICP-MS data from the current study showed low correlation with the prior ICP-OES data (Table 4). ICP-MS is known to be more sensitive than ICP-OES. ICP-MS detects concentration ranges as low as ng/g whereas ICP-OES is limited to detection in the μg/g range [13]. ICP-MS also has a broader range of detection than ICP-OES. While ICP-MS can detect differences in a single sample up to 12 orders of magnitude, ICP-OES is limited to detecting up to 6 orders of magnitude in a single sample [13]. Minerals for which positive correlations were observed between the two datasets were the more abundant elements detected, while negative correlations were mostly detected for elements in low abundance in the species. Moreover, we found that ICP-MS data for the overlapping accessions showed differences in five of nine elements that represent the extreme high and low levels of mineral accumulation. These data are consistent with the ideas that ICP-MS has both greater range and sensitivity than ICP-OES. We suggest that differences in element detection method had a significant contribution to the difference observed for overlapping accessions.

Genetic and environmental variation are also likely contributors to differences observed between the ICP-OES and ICP-MS data. Of the 30 accessions compared, 20 of the ICP-MS accessions are derived from single plant selections of germplasm sampled in bulk pools for profiling by ICP-OES. Visible segregation of plant phenotypes was observed for a subset of the accessions profiled by ICP-OES. In addition, the published ICP-OES and our ICP-MS survey used seed lots produced from distinct soil types. Seed ionome concentrations are influenced by soil type and environment [7, 14, 33, 44, 45]. Pea production in the United States of America is primarily located in the northwest and upper Midwest, with Washington state being the largest producer in 2018 [46]. The peas in this study were propagated through the USDA-ARS Western Regional Plant Introduction Station in production-relevant soil types and environments. However, a significant limitation of the data is that each accession was produced during a single field season and in a single soil type. The specific levels of minerals for accessions of interest should be confirmed in multiple soil-types prior to use in a breeding program or quantitative genetic analysis. These results do provide improvements over the prior ICP-OES data, which are an average of two technical replicates of seeds harvested from plants grown in potting soil and vermiculite in a greenhouse [27]. Therefore, the seed nutritional content reported here is likely to be more relevant to one of the major production regions.

Pea seeds have enormous potential as a sustainable, nutrient dense, plant-based source of nourishment as part of a diverse human diet [19, 26, 47]. With the human population worldwide continuing to expand, and the prevalence of micronutrient malnutrition already remarkably high, improving the nutritional value of crops, and pulses especially, is a priority [2, 48, 49]. Three clusters of accessions were identified in this study with high mineral nutritive value. Only four of the fifteen accessions in these clusters were represented in prior ICP-OES data [27]. One accession, PERFECTION, ranks above average for the majority of minerals in both datasets suggesting that this accession is likely a source of genetic variation for increased mineral content. The three additional overlapping accessions scored below ICP-OES averages for 3–7 minerals each. However, single plant selections were analyzed by ICP-MS for PANIA and PATEA, making these genotypic comparisons indirect, while CENTRALI-SIBIRICUM accumulated higher levels of Cu in our ICP-MS survey.

## Conclusion

The ionomic analyses presented here show positive relationships between mineral micronutrients in pea seeds. Clustering of element accumulation phenotypes identified fifteen accessions with above average mineral nutrient content. These accessions would need to be validated as having a heritable increase in mineral content prior to incorporating into biofortification efforts. The comparison of historic ICP-OES elemental profiling and the ICP-MS data in this study suggests that ICP-OES phenotypes are less accurate for K, Fe, Mn, B, and Cu. Further phenotyping of germplasm resources is likely to identify additional heritable variation in mineral nutrient content.

## Supporting information

S1 Table Elemental concentrations of 96 pea accessions obtained from ICP-MS. (XLSX)
